# Analyzing risk contagion and volatility spillover across multi-market capital flow using EVT theory and C-vine Copula

**DOI:** 10.1016/j.heliyon.2024.e39918

**Published:** 2024-10-29

**Authors:** Fahim Afzal, Haiying Pan, Farman Afzal, Rana Faizan Gul

**Affiliations:** a Business School of Hohai University, Nanjing, 211100, China; b Institute of Business and Management, University of Engineering and Technology, Lahore, Pakistan

**Keywords:** Volatility spillover, Risk contagion, EVT theory, C-vine Copula

## Abstract

There exists a potential interdependence among the United States markets, alongside an exceptional dependence on the East Asian stock markets. This transmission of risks is similarly evident in funds that are traded within markets. The current study seeks to uncover the pathways of risk contagion among various financial markets. This study examines how risks of funds spread across borders among China, the United States, and East Asia using Extreme Value Theory (EVT) and C vine copula quantile regression. It uses models such as AR (1), EGARCH (1, 1), Peak over Threshold (POT), and Copula methods to predict volatility events and correlation patterns during times of high volatility versus regular periods. Notably, the United States exhibits a risk transmission effect on the East Asian market compared to China. Furthermore, the findings indicate that, in times of high volatility, the risk spillover effect is comparatively weak, which is contrary to the situation of the US market. The study suggests that China's financial impact could progressively rise due to initiatives aimed at sector integration and enhancing financial independence. These findings have enlightening consequences for macro-prudential regulatory agencies, emphasizing the necessity of effective regulation to address cross-border risk spillovers. International investors can benefit from these results by incorporating risk-hedging strategies, accurately evaluating derivatives, and making wise investment decisions.

## 1 Introduction

In the crisis, the proliferation of investor anxiety initiated an influx of trading activity, resulting in considerable risk transmission among interconnected financial resources [1]. When a specific stock market is in trouble, extreme price fluctuations caused by market dependence may lead to significant losses in the relevant market. This phenomenon is referred to as risk spillover, which may appear indirectly via direct contractual links and strengthened counterparty credit risk, in addition to monetary effects and liquidity spirals [2].

Accurately predicting a stock market crisis presents significant challenges, yet these crises frequently accompany considerable volatility. Extreme price changes during periods of volatility may disrupt the stability of cross-border financial markets. It is worth noting that volatility clusters, a well-known fact of financial stylization, indicate the persistence of excessive price changes, i.e., high variability of returns. Consequently, the risk exposure of a stock market has increased significantly for a long time due to the risk spillover of a similar stock market, which poses a significant risk to the overall stability of its financial market [3].

Like other Asian emerging stock markets, the Chinese stock market has evolved within the last decade into an essential component of foreign investment managers' diversified risk investment portfolios. This approach aims to enhance profitability while simultaneously mitigating risks. With the increasing globalization of Asian stock markets and the booming development of the mobile internet, important information is rapidly spreading in various markets, leading to unprecedented strong cooperation with other stock markets. At the same time, China is developing its economic and financial relationships with other countries by adapting to market modernization, such as the stock connect plan.

Implementing various policies to liberalize China's financial industry has significantly boosted the nation's influence in Asian stock markets. Numerous research studies have yielded noteworthy results, illustrating that China's efforts to modernize its financial sector have substantially impacted the market efficiency, interdependence, and susceptibility to risk [4,5]. Additionally, there exist notable disparities in the dynamics between the Chinese stock market and the HK (Hong Kong) stock market, both starting and after the introduction of the stock channel [[6], [7], [8], [9]]. Consequently, due to the growing integration of Asian stock markets, the diversification benefits of including Chinese stocks in international investment portfolios may diminish. Similarly, the stock markets of other Asian countries may become more susceptible to the influence of Chinese equity markets.

In recent years, two stock market crises, such as the stock market crash in China in 2015 and the downfall of the United States stock market in 2020, have garnered academic interest. Numerous researches have been conducted to investigate the disaster that occurred in 2015 in the Stock Market of China [[9], [10], [11], [12]]. Similarly, there is an increasing collection of research focusing on the stock market crash in the United States during the global epidemic breakout [[13], [14], [15]]. Asia, intimately linked to both China and the United States in commercial and financial activities, presents some contrasts. Asian countries have many things in common with China, like strong trading in stock synchronization, close physical proximity, comparable meteorological conditions, and interconnected regional cultures. Similarly, commercial and monetary collaboration between Asia and China has increased dramatically, while the correlation between the United States and Asia seems to weaken in recent years. Nevertheless, in the context of the turbulent era marked by the market crisis, there remains a notable lack of investigation into the potential spillover effects of the Chinese and US stock markets on the stock markets across Asia. In addition, little research has been done on China's influence on the Asian market's emergence. Therefore, studying the spillover effects of these differential risks on the stock markets of Asia is of great significance and depth.

According to the perspective of dependence, the democratization of China's market has expedited the convergence trend of Asian stock markets. In the years 1990 and 2016, the dynamic correlation of stock returns in the United States and Asia increased from 10 % to 50 % [16]. The market turbulence faced by these two countries will undoubtedly foster strong market connections and lead to considerable risk exposure in Asia's stock markets. Therefore, cross-border risk mitigation must research how China and the US affect risk in Asian stock markets. A risk management department's understanding of the structural disparities in its reliance on Asian stock markets and investors' analysis of the effects of the largest developed and growing stock markets can be aided by analyzing these risk spillovers.

The US dollar is critical to international trade and asset valuation since it is the world's reserve currency. Additionally, the US has invested substantial money in the Asian market. Due to these considerations, risk spillovers from America and China will likely influence Asian financial markets. Risk spillovers of the Chinese and American stock markets have hurt Asian economic activity and financial development, and they could produce financial instability globally. Asian stock markets and global financial markets are intertwined. To keep the economy stable and help stakeholders modify their investment portfolios, risk management departments should consider the practical implications of evaluating the spillover impacts of these risks on the stock markets of Asia.

The Chinese and American stock markets may significantly impact East Asian markets despite having experienced severe volatility during market crises. The US stock market is well-positioned worldwide and substantially impacts the development of the world's financial systems. This advantage is explained by the US dollar's dominance in international trade and investment and good financial stability, which has resulted in dangers to the US stock market's decline that impact Asian stock markets.

In contrast, the financial market of China varies greatly. Despite ranking as the second biggest market globally, it is still a developing market with unique characteristics. Excessive speculation remains a common trouble in the Chinese financial market. The dependence between the Chinese and the Asian stock market is established on their trade, commerce, and economic relationships, as well as the common characteristics of the region. In this regard, two interesting questions arise. Firstly, what are the differences in the impact of these two markets on the Asian Stock markets? Secondly, will the Chinese and United States stock markets conditionally spread to East Asian stock markets under certain conditions? These issues will be discussed in the empirical section.

In financial risk management, most literature is found while analyzing the volatility of stock returns across different financial markets. Various financial contagion models are developed using Garch families or other multivariate models. A lack of literature exists in addressing the market behavior of funds, which is highly importance for investors interested in funds. Therefore, this study addresses the financial contagion and volatility in funds. In addition, the use of Extreme Value Theory (EVT) theory and cupula models makes this approach more sophisticated for addressing the volatility in funds. The challenging component of this study is creating a reliance framework that incorporates not only Chinese and US stock markets but also all other Asian markets. Given this intricacy, the major objective of this essay is to build a reliance framework between the stock markets of America and China, using Japan, South Korea, and Hong Kong as exemplars of East Asian stock markets. This study aims to address existing gaps and contribute additional insights to the existing body of knowledge. Initially, we applied advanced techniques that various experts had previously implemented to detect potential variations in the variance structure of stock returns. Notable studies by Ewing and Malik (2016) [17], Hood and Malik (2018) [18], and Rapach and Strauss (2008) [19] Served as our foundational references. We assessed the duration of the notable volatility period before and after the market crisis to determine the extent of disruption. In cases of extreme market events, we followed the approach proposed by Koliai (2016) [20] and leveraged EVT to simulate how the distribution of the return series behaves in its tail.

We constructed models to capture the interdependencies among China, the United States, and East Asia stock markets. This was accomplished using the method known as C-vine Copula. In assessing the risk of contagion from the stock markets of the USA and China to the Asian (East) stock markets during periods of high volatility period, we employed the Conditional Value at Risk (CoVaR) methodology. Quantile regression techniques were applied with consideration of the projected C-vine dependency structure, drawing insights from the work of Fang et al. (2021) [21] and Shahzad et al. (2018) [22].

Lastly, in line with previous scholarly practices, we subjected our findings to a bootstrap Kolmogorov-Smirnov test to validate the robustness of spillover effects. This evaluation procedure, as employed by Bernal et al. (2014) [23] and Shahzad et al. (2018) [22] was integral to ensuring the reliability of our results. We scrutinized the variations in risk outcomes based on these insightful empirical findings.

This article's remaining section is organized so that the next section reviews relevant literature. The following sections, 3 and 4, give a full description of the econometric techniques and data. Section 5 discusses risk spillovers through the lens of EVT theory and copula quantile regression, while the last section presents the conclusion.

## 2 Literature review

Nowadays, researchers are primarily dedicated to regularly analyzing the complex patterns of stock market volatility. Notably, the investigations undertaken by Bollerslev (2006) [24], Nelson (1991) [25], and Glosten et al. (1993) [26] Have made significant contributions to the specific field of research. These studies specifically developed methods to investigate the “leverage effect,” a phenomenon that prior historical conditional covariance models overlooked. The leverage effect is crucial in comprehending the asymmetric volatility of stock market returns. The stock market variance, unaffected by individual conditions, is instead impacted by the economic and political circumstances of the country, including government restrictions, foreign shocks, and economic cycles.

### 2.1 Literature on volatility spillover

Extensive research in the field has revealed the effects of structural changes on the day-to-day fluctuations in stock market volatility. Initial studies concentrated on analyzing the conditional covariance rather than investigating the underlying factors that cause substantial shifts in the stock market. Researchers Banerjee and Urga (2005) [27], Caporin and Malik (2020) [28], Ewing and Malik (2016) [17], Hillebrand (2005) [29], Rapach and Strauss (2008) [19], have focused on their research on comprehending the impacts of negative events during the structural changes in stock markets. Their studies have shown significant results in explaining the superiority of Value at Risk (VaR) over older methodologies that neglected the long-lasting disruptions induced by structural changes in stock markets.

Stock market researchers are prioritizing their efforts to comprehend the causal relationship between structural changes in one market and the subsequent influence on the performance of other markets. To understand this issue, many models have been used to clarify the complicated relationships between stock markets.

Analysts rely on copulas to assess how stocks perform by examining the relationships between them. Researchers utilize bivariate copulas to analyze correlations among commodities, as highlighted in studies by Kumar et al. (2020) [30] and Ouyang et al. (2022) [31]. These scholars primarily use bivariate copulas to unravel the ties within stock relationships. Shahzad et al. (2018) [22] research provides insights into these connections, particularly shedding light on the interplay between Chinese and global stock markets through applying bivariate copula and EVT.

### 2.2 Complex risk contagion paths

The relationships between stock markets play a role in determining the interdependence and complexity of the system. Joe (1996) [32] has proposed a method for evaluating the structure of stock markets by transforming multivariate copulas into bivariate ones. Likewise, Bedford and Cooke [33] have contributed to their introduction of vine copulas, elucidating its framework. The illustration of vine structures by these researchers underscores the hidden patterns and interconnected relationships present within a multivariate system.

Weiβ and Supper [34] believe that vine copulas are a highly effective technique for imitating the complex relationships between shares on the NASDAQ stock exchange. The effectiveness of financial modeling performed by stock market experts was improved. In addition, Koliai [20] led a significant change in approach through their innovative contributions to defining the concept of stress tests. This study investigated the complex interrelationships and linkages among five prominent stock markets, six currencies, and commodities. In addition, Xiao [9] investigated the complex features of risk transmission in Asian stock markets using vine copula methodologies. Their study provides a comprehensive description of the process behind stock market correlations.

The main focus of the study of Jian et al. [35], is the transmission of stock market volatility within the framework of conditional risk. Generally, two approaches are used to study the spread of volatility. The researchers who developed the quantile regression approach have achieved exceptional success. This strategy thoroughly examines risk transmission under certain conditions across different quantiles.

#### 2.2.1 Dynamic conditional correlation model (DCC)

In 2017, the research employed a Dynamic Conditional Correlation (DCC) model based on Garch. It is crucial to analyze the changing relationships between different types of investments to understand how risk is transmitted between asset classes. This technique enables such an investigation [35,36]. Volatility spillovers in risk networks were identified by applying the quantile approach, which helped determine the dual origins of these spillovers. The study on stock market volatility spillovers mostly relies on the conditional risk paradigm established by Jian et al. [35]. Researchers frequently employ two methodologies regarding volatility spillovers. Quantile regression, introduced by these researchers, is a widely used and successful approach. This approach thoroughly analyses the conditional risk spillovers across different quantiles. Furthermore, Brownlees and Engle (2017) [37] and Acharya et al. (2017) [38] employed a Garch-based DCC model. This approach facilitates the analysis of dynamic correlations among various asset classes under assessment, which is crucial for comprehending risk transfer from one asset class to another.

#### 2.2.2 Conditional value at risk for measuring volatility

The term “Tail Event-Driven Networks” (TENET) was introduced by Yu, Hardle, and Wang [39]. Their quantile regression investigation of systemic risk in U.S. corporations primarily examined the relationships identified by Conditional Value at Risk (CoVaR). Wang et al. [40] employed quantile regression to investigate the transmission of risk among Chinese stock markets. Despite this, applying quantile regression with CoVaR poses a unique set of challenges since it depends on three crucial aspects for analysis: macro indicators, stock prices, and the company's balance sheet being studied. Maintaining the intricate connections among financial assets and ensuring alignment within the economic system can present significant challenges and difficult pledges. When the systemic risk is present, the traditional quantile regression approach looks ineffective in collecting high-frequency data information since it only addresses linear correlations across variables.

Adrian and Brunnermeier [2] have employed the quantile technique to analyze risk networks and illustrate the dual mechanisms of volatility spillovers. In their prominent paper, Härdle, Wang, and Yu (2016) [39] introduced a method called TENET. Their study employed quantile regression to examine the systemic risk across US enterprises, focusing on their interconnectedness as measured by CoVaR. Wang et al. (2018) [40] employed quantile regression to analyze risk transmission between the Chinese stock markets.

Nevertheless, implementing quantile regression using CoVaR presents distinct challenges due to its reliance on three essential components for analysis: macro indicators, stock prices, and the firm's balance sheet under examination. Aligning the financial system and keeping the complex relationships of financial assets up to date may be intimidating. Traditional quantile regression addresses linear relationships among variables; hence, the model appears inadequate in capturing high-frequency data information when systemic risk is present [41].

#### 2.2.3 Measuring risk paths and spillover using copula method

The model based on stock market data offers an approach to understanding relationships between different factors. This method uses the copula technique, which helps model the relationship structure and time series returns separately based on scenarios. On the side, the model created using data from the stock market provides a way to understand the relationships among numerous components. The copula method is often used to analyze the relationships between time series returns and their dependence structure. The methods of application can differ depending on the situation. Wang et al. [40] introduced copula regression as a way to illustrate the relationship between return series by focusing on quantiles. Employing this technique allows for an adaptable assessment of risks associated with risk transmission among different structures dependent on return series. This approach enables an evaluation of interconnectivity susceptibility to risks and intricate relationships among assets. Both referenced studies by authors [37,38] analyze risk transfer mechanisms and complex structures across stock markets. It is crucial to emphasize the importance of considering events and contingent situations. To capture the two-way link between return series using quantiles, Bouyé and Salmon (2009) [42] introduced the copula regression technique. This approach provides a thorough way to grasp the risk transfers among interconnected return structures. This method can give insights into the asset class relationships, interdependencies, and systemic risks. Fang et al. (2021) [21] and Jiang et al. (2021) [43] delve into examining networks and risk transfers among stock markets. Their research emphasizes the importance of both unconditional situations in their analysis. Moreover, their findings align with studies that have closely explored relationships and risk transmissions between Chinese stock markets and other global markets within this financial framework. Jiang et al. (2021) [43] effectively analyzed the Chinese, US, Japanese, and British stock market risk using a similar methodology. This reinforces the foundation of this methodology as applied by earlier academics.

Furthermore, research has explored the complex links and risk transmission between Asian stock markets and North American markets throughout this interconnected financial system. A detailed study is carried out to investigate and evaluate the risk linked with the stock markets of the United States of America, China, Japan, and the United Kingdom using a comparable methodology. In this way, the foundation of the approaches that were used in earlier investigations is strengthened [38,44].

## 3 Econometric methodology

This study employs econometric methods to measure and analyze risk spillovers between markets. The selected methods—E-GARCH with structural breaks, EVT, the Copula method, and Spillover detection—are particularly well-suited for capturing different dimensions of market risk and their interconnections. The rationale behind these methods lies in their complementary strengths: the E-GARCH model addresses volatility clustering and asymmetric effects; EVT focuses on extreme events; the Copula method captures dependencies between markets; and Spillover detection reveals risk transmission across markets.

Inappropriate component evaluation in the GARCH method can result from disregarding a structural modification in the indefinite variance, according to Hillebrand (2005) [29]. To assess the hypothesis of a variance break against the theory of constant unconditional variance, Inclán and Tiao (1994) [45] introduced the IT statistic, which calculates the cumulative sum of squares. Our study presents a nonparametric correction utilizing the Bartlett kernel and applies the adjusted IT statistic. Furthermore, we enhanced the variance equation of the model of conditional variance by incorporating the detected underlying structural breaks. Nelson (1991) [25] introduced the Exponential Generalized Autoregressive Conditional Heteroscedastic (EGARCH) model to accommodate present asymmetric volatility shocks. The EGARCH(1,1) model is defined as follows in equations (1), (2)):

(1) $$r_{t}=\mu +\rho r_{t-1}+\varepsilon_{t},\varepsilon_{t}=h_{t}z_{t}$$

(2) $$\ln\left(\;h_{t}^{2}\;\right)\;\omega +\alpha \left[\left|z_{t-1}\right|-E|z_{t-1}|\right]+\gamma z_{t-1}+\beta \;\ln\left(h_{t-1}^{2}\right)$$

μ represents the mean and the conditional variance, zt is considered an independent casual variable. The parameter α of the magnitude effect, indicating a substantial influence on volatility. In contrast, the parameter γ serves as a test for the sign effect, accentuating asymmetric influences related to volatility. Positive changes in volatility resulting from favorable news are reflected by the total α + γ, depending on whether the innovation succeeds εt surpasses zero. Conversely, negative changes in volatility corresponding to unfavorable news are represented as α − γ when the innovation εt falls below zero.

### 3.1 EGARCH model with a structural break

This model having breaks is defined as in equations (3), (4)):

(3) rt=μ+ρrt−1+εt,εt=htzt

(4) ln(h2t)=ω+d1D1+…….+dnDn+α[|zt−1|−E|zt−1|]+γzt−1+βln(h2t−1)

By the seminal works of Ewing and Malik (2010) [46] as well as Rapach and Strauss (2008) [19], the present study considers a set of dummy variables denoted as D_1_ through Dn. These variables are assigned a value of unity to signify instances in which the variance process becomes linked to a structural alteration.

### 3.2 Extreme value theory

The Peaks Over Threshold (POT) methodology derived from EVT is employed to analyze the model within the scope of our investigation. The concept of the excess distribution beyond a specified threshold is outlined as follows in equation (5):

(5) $$F\eta \;\left(z\right)=P\left(Z\;–\;\eta \leq \;z|Z\;>\;\eta \;\right)=\frac{F\left(z+\eta \right)\;–\;F\left(\eta \right)}{1\;–\;F\left(\eta \right)}$$

The Generalized Pareto Distribution (GPD) demonstrates asymptotic behavior as applied to a high threshold of sequential innovations. An empirical cumulative distribution function complements the intermediate segment of the overall distribution.

### 3.3 C-vine Copula models

Using a vine copula is a valuable approach for examining intricate interrelationships encompassing multidimensional variables. Specifically, the C-vine copula elucidates distinctive correlations within multivariate scenarios, wherein the interdependence between two variables is contingent upon the influence exerted by an intermediary connecting variables. The concise elucidation of the density function of the four-dimensional, for the C-vine, is delineated as follows in equation (6):

$$f\left(z_{1},z_{2},z_{3},z_{4\;}\right)=\prod_{a=1}^{4}f_{a}\left(z_{a}\right)\prod_{b=2}^{4}c_{1,b}\left(F_{1}\left(z_{1}\right),F_{b}\left(z_{b}\right)\right)$$

(6) $$\times \prod_{j=2}^{4-1}\prod_{i=1}^{4-j}c_{j.j+i|1,\ldots \ldots \ldots \ldots j-1\;}\left(F\left(z_{j|z_{1},\ldots \ldots \ldots .,z_{j-1}}\right),F\left(z_{j+1|z_{1},\ldots \ldots \ldots .,z_{j-1}}\right)\right)$$

The conditional copula function is represented as (Cj, j + i|1, …,j- 1). We use the previous approach based on the maximum spanning tree to maximize the sum of absolute dependences to estimate the C-vine [47].

It is described as in equation (7):

(7) $$\max\;\sum_{e=\left\{i,j\right\}}\left|\delta_{ij}\right|,1\leq i,j\leq N$$

here, e shows an edge. δij was used to calculate the bivariate dependence of the nodes connected to the edges.

#### 3.3.1 Copula quantile regression

Applying a copula framework to quantile regression involves the characterization of a linear association between the reaction and the predictor across diverse percentage levels, as pioneered by Koenker and Bassett (1978) [48]. The estimation of quantile regression coefficients necessitates the resolution of the subsequent optimization task presented in equation (8):

(8) $$\min_{\beta \epsilon R^{k}}\left\{\left(\sum_{t=1}p-I_{\left\{y_{t}\leq x_{t\beta }\right\}}\right)\left(y_{t}-x_{t}\beta \right)\;\right\}$$

### 3.4 Measuring risk spillovers

The C-vine copula, as indicated in the referenced study, uncovers a fundamental linkage connecting distinct nodes within the system. This dependency relationship, documented by the C-vine copula, illustrates a central connection between the nodes. The derived conditional copula quantile equation can effectively measure the extent of risk spillovers between these interconnected markets. The ensuing definition delineates the conditional copula quantile formula as given in equation (9), which is a pivotal tool in our analysis.

(9) $$CoVaR_{\tau }^{y|x_{1}={\hat{F}}_{x_{1}}^{-1}\left(\beta \right)}={\hat{F}}_{Y}^{-1}\left(\;{\hat{C}}_{V|U_{1}}^{-1}\left(\tau |\beta \right)\;\right)$$

where ${\hat{F}}^{-1}$,*X*_1_ (*β*) = inverse distribution function of *X*_1_. (The market)*X*_1_ is in a turmoil state if *β* takes $\hat{F}$ *X*_1_(*VaR*_1_ *β*). (On the contrary, the market)*X*_*1*_ is in a normal state if *β* takes $\hat{F}$ *X*_1_ ($x_{1}^{0.5}$), for $x_{1}^{0.5}$ is the median value of *X*_1_.

Similar to this, the risk spillovers from one market to the others are as follows in equation (10):

(10) $$CoVaR_{\tau }^{y|x_{1}={\hat{F}}_{x_{1}}^{-1}\left(\beta \right)}={\hat{F}}_{Y}^{-1}\left(\;{\hat{C}}_{V|U_{1;}U_{2}}^{-1}\left(\tau |{\hat{C}}_{U_{1}|U_{2}}^{-1}\left(\beta ,u_{2}\right)\right)\;\right),\beta =F_{X_{2}}\left(\;VaR_{\beta }^{2}\right)$$

The impacts of risk spillover from one stock market to another are finally estimated using Adrian and Brunnermeier (2016) [2] relative technique as given in equation (11).

(11) $$\Delta CoVaR_{\tau }^{y|x_{1}}=\frac{\left(\;CoVaR_{\tau }^{y|x_{1}={\hat{F}}_{x_{1}}^{-1}\left(\beta \right)}-CoVaR_{\tau }^{y|x_{1}={\hat{F}}_{x_{1}}^{-1}\left(x_{1}^{0.5}\right)}\;\right)}{CoVaR_{\tau }^{y|x_{1}={\hat{F}}_{x_{1}}^{-1}\left(x_{1}^{0.5}\right)}}$$

The locations where the formulae evaluate related spillover effects. In order to evaluate the effects of spillover, a procedure is used which is used in extensive investigations [22,23]. To determine the importance of the effects of spillover, we use the bootstrap Kolmogorov-Smirnov (KS) test as given in equation (12), which was created by Abadie (2002) [49].

(12) $$KS_{mn}={\left(\;\frac{mn}{m+n}\right)}^{\frac{1}{2}}sup_{x}\left|F_{m}\left(x\right)-G_{n}\left(x\right)\right|$$

where the distribution functions of the cumulative CoVaR are in the heightened and normal condition, respectively, are denoted by Fm(x) and Gn(x). The two samples have a total number of m and n.

## 4 Data descriptions

The particular stock market indices encompass the FTSE 100 index (UK), the SSEComp index (China), the S&P 500 index (US), the Hang Seng index (Hong Kong), and the KOSPI Composite index (South Korea). These indices, sourced from Yahoo Finance, span from July 6, 2010, to June 29, 2023, reflecting market fluctuations. The last-observed-carried-forward (LOCF) methodology is employed to address data gaps. Notably, a distinct approach is adopted for the S&P 500 index's closing price to mitigate the influence of asynchronous trading effects. The calculation of stock index returns follows the formulation: 100 (lnxt ln xt 1), with xt denoting the opening price on day t.

**Fig. 1:**
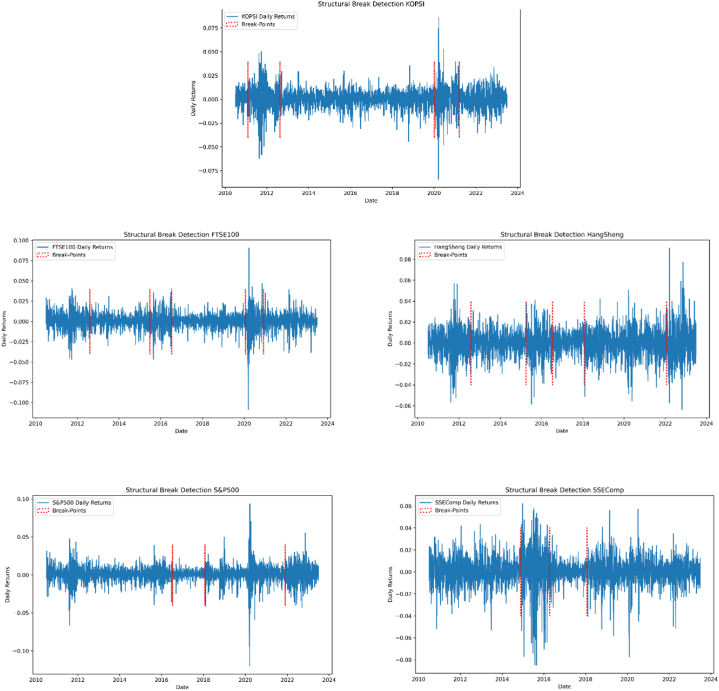
Stock indexes, standard deviation and structure breakdown.

**Fig. 2A:**
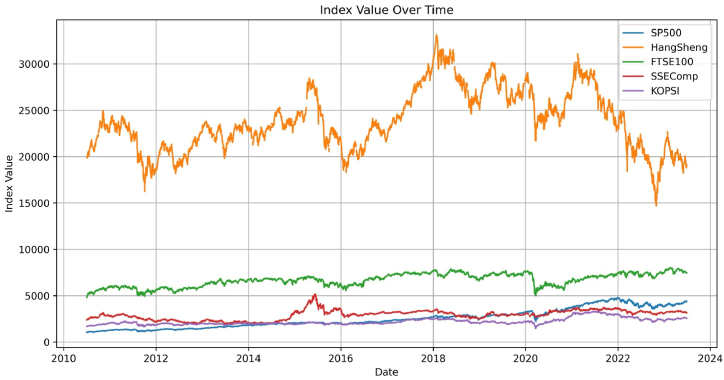

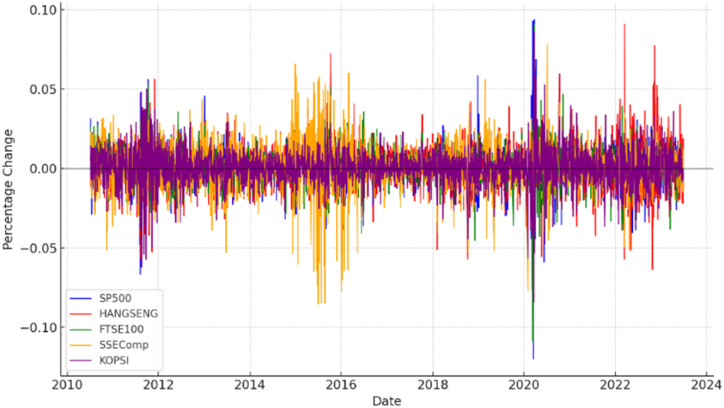
Relative stock prices over a crisis period. Percentage change of returns over a crisis period.

The updated ICSS algorithm is employed in Table 1 to identify key structural junctures in the underlying volatility of stock markets. The illustrative index returns displayed in Fig. 1 portray the distinctive attributes characterizing volatility within the stock market. The delineated segments highlight two anomalous periods marked by notable volatility clustering. Notably, the periods of heightened volatility in the US stock market, UK stock market, China's stock market, Hong Kong Stock market, and Korean stock market surrounding the crisis are demarcated by crimson borders on the left and right, respectively. The Chinese stock market crisis of 2014 unfolded during escalated volatility within the Chinese stock market. Over three months, the SSEComp index experienced a steep decline of nearly 2000 points, plummeting from 5353 to 3354 (Fig. 2A and B). A pervasive trend of heightened volatility across various financial markets marks the prevailing juncture. The notable correlation between the Hong Kong stock index and the Chinese stock market raises the possibility of the Chinese market exerting influence on other stock markets through the Hong Kong platform. The global impact of the COVID-19 pandemic, coupled with the heightened volatility in the US stock market, has induced widespread apprehension. During this period, the downward spiral of various stock markets due to panic-driven shocks has been exacerbated by the abrupt decline in the US stock market.

**Table 1:** Structural breaks in volatility.

Stock Index	BP1	BP2	BP3	BP4	BP5
SP500	July 11, 2016	January 26, 2018	November 24, 2021		
HangSheng	August 1, 2012	March 31, 2015	July 13, 2016	January 25, 2018	January 24, 2022
FTSE100	August 9, 2012	June 24, 2015	July 11, 2016	January 23, 2020	December 3, 2020
SSEComp	November 25, 2014	April 12, 2016	January 25, 2018		
KOPSI	February 8, 2011	August 17, 2012	January 3, 2020	March 16, 2021	

**Note:** This table presents the structural breakpoints of each selected stock during the study timeframe. BP1-BP5 shows each stock's Break-Point date.

The response to the pandemic in China has led to a noticeable slowdown in the transmission of infections, which is particularly reflected in the stock market trends. Significantly, China's stock market began to show indications of recovery from January 21, 2020, which coincided with the onset of the US stock market's downturn. Nonetheless, the resurgence in the Chinese market was exhibited only briefly before stalling alongside the continuous decline in the US stock market. The significant repercussions stemming from the decline of the US stock market are undeniably exerting an influence on the stock market in China. The prevailing circumstance of exceptional volatility gives rise to heightened sentiments that pervade all stock markets. This juncture implies a heightened propensity for greater and more widespread interconnectedness across global stock markets compared to the prior phases characterized by elevated volatility in the Chinese stock market. In contrast to these two distinct periods, the present era displays greater stability. The forthcoming empirical analysis will encompass all these temporal segments to facilitate a comprehensive comparative assessment.

Table 2 presents statistical summaries of stock index returns observed over distinct periods. The presence of noteworthy statistical dispersion becomes apparent when the mean return closely approximates zero, accompanied by a substantial standard deviation. The pronounced left-skewed distribution of all index returns suggests that dynamic volatility could lead to abrupt declines, potentially exacerbated by inherent leverage effects. Notably, the degree of kurtosis exhibited by the US stock market surpasses that of the comparative stock indices. This heightened kurtosis signifies a broader tail in the distribution and implies a heightened potential for significant fluctuations in stock prices. The occurrence of substantial minimum values is indicative of extreme losses. This study presents notable statistical insights into the dynamics of return series and the extent of extreme downside risk, explicitly focusing on periods of high volatility within the Chinese and US stock markets.

**Table 2:** Descriptive statistics for index returns.

	FTSE100	SP500	HANGSENG	SSEComp	KOPSI
Min	−0.1087	−0.1198	−0.0636	−0.0852	−0.0839
Max	0.0905	0.0938	0.0908	0.0784	0.086
Mean	0.0002	0.0006	0.0001	0.0002	0.0002
Std.dev	0.0106	0.0116	0.0133	0.0136	0.0109
Skewness	−0.4792	−0.3803	0.1131	−0.6035	−0.1929
Kurtosis	8.8212	12.3486	3.6199	6.1444	6.4572
ADF	−16.3227	−17.85	−53.9222	−11.3843	−35.5767
Jarque-Bera	9418.383	18310.52	1573.611	4690.6	5005.626
PP	−55.155	−62.1067	−54.0013	−52.7983	−53.9845
Q2(20 %)	−0.0061	−0.0057	−0.0089	−0.0077	−0.0067
Arch(20)	0.177	0.000158	0.09731	0.441	0.09862
VaR(0.05)	−0.0169	−0.0178	−0.0213	−0.0194	−0.0169
VaR(0.01)	−0.0314	−0.0328	−0.0367	−0.0412	−0.0321

**Note:**Table 2 presents statistical summaries of stock index returns observed over distinct periods. Key stats such as standard deviation, ADF, and Jarqu-Bera tests are included as prerequisites for further analysis. Moreover, value at risk at different quantiles, such as 5 % and 1 % degrees of freedom, has been estimated.

A comparative analysis is undertaken to discern disparities between overall market performance and high-volatility phases. These minimal values emerge from two instances of heightened volatility (see Table 3). Empirical data reveals that China's stock market demonstrates heightened downside risk during phases of elevated volatility compared to relatively stable periods. A parallel pattern of high-volatility intervals in the US stock market is observed. Similar trends are corroborated by the statistical analysis of downside Value at Risk (VaR) values. The influence of risk spillages from the largest stock markets, China and the US, reverberates across East Asian stock markets.

**Table 3:** Descriptive statistics during the high volatility period.

	SP500	HangSheng	FTSE100	SSEComp	KOPSI
# High Volatility period of US					
Min	−0.04157655	−0.06385702	−0.03918789	−0.05141732	−0.03556177
Mean	−0.00107648	−0.0009967	−0.00035808	−0.00049692	−0.00072682
SD	0.01352178	0.0189814	0.0097476	0.01048034	0.0112725
VaR.0.05.	−0.02226386	−0.02616178	−0.01907138	−0.01713508	−0.01905968
VaR.0.01.	−0.03310139	−0.04507573	−0.03308809	−0.02615081	−0.02702247
# High Volatility period of China					
Min	−0.04245396	−0.05856722	−0.04892175	−0.08601495	−0.02749599
Mean	−0.00067536	−0.00054968	−0.00082002	0.0007907	−0.00025428
SD	0.00939707	0.01230269	0.01020464	0.02091654	0.00782775
VaR.0.05.	−0.01646833	−0.01843346	−0.01763126	−0.03400547	−0.01267073
VaR.0.01.	−0.0263424	−0.03111305	−0.02996158	−0.07375216	−0.02055374
# High Volatility period of UK					
Min	−0.11223337	−0.05599969	−0.11336335	−0.07699987	−0.08485783
Mean	−7.7558E-05	−0.00061448	−0.001816	0.00051734	0.00076834
SD	0.02427172	0.01708363	0.02114382	0.01501924	0.02027924
VaR.0.05.	−0.03669977	−0.02353979	−0.0376938	−0.01881008	−0.03578054
VaR.0.01.	−0.07998135	−0.05006503	−0.05805863	−0.03947427	−0.05253561
# High Volatility period of HongKong					
Min	−0.11223337	−0.05751304	−0.11336335	−0.07699987	−0.08485783
Mean	−0.00027433	−0.00049831	−0.0005498	−5.3166E-05	−0.00010952
SD	0.01383181	0.01334522	0.01210951	0.01215003	0.01260899
VaR.0.05.	−0.02206213	−0.02238343	−0.01841613	−0.01836827	−0.01936337
VaR.0.01.	−0.04237768	−0.04101235	−0.0396562	−0.03709329	−0.03908601
# High Volatility period of Korea					
Min	−0.11223337	−0.05599969	−0.11336335	−0.07699987	−0.08485783
Mean	7.7743E-05	−0.00022498	−0.00108616	0.00028965	0.00113434
SD	0.02098084	0.01618968	0.0184505	0.01382196	0.01858709
VaR.0.05.	−0.03362625	−0.0244247	−0.03343321	−0.01916945	−0.03144522
VaR.0.01.	−0.06617673	−0.0449472	−0.0495511	−0.03781816	−0.05013257

**Note:**Table 3 presents the statistics during the selected stocks' high volatility periods. As shown in this table, a parallel pattern of high-volatility intervals in the US stock market is observed. The statistical analysis of downside VaR values corroborates similar trends. The influence of risk spillages from the largest stock markets, China and the US, reverberates across East Asian stock markets.

Compared to a standard normal distribution, all the index returns in the dataset consistently show significant characteristics that suggest they are not normally distributed. When we use Jarque-Bera tests to analyze the returns, they indicate that they don't follow a normal distribution. Additionally, we conducted Augmented Dickey-Fuller (ADF) tests at a significance level of 1 %, and the results provide strong evidence to reject the idea that there is a long-term trend (unit root) in all the index returns. This finding is further confirmed by the Philips Perron (PP) test. Furthermore, the empirical results ascertain ARCH effects in the index returns series. Both the Q2 (20) and ARCH (20) tests show significant results at the 1 % significance level, suggesting that there is conditional heteroscedasticity in the returns, meaning that the volatility of returns varies over time.

Table 4 presents the linear interdependence observed within the stock markets. Notable variations in correlation coefficients are identified. Notably, except for the US with China, Hong Kong & South Korea, and China with the UK, the correlations linking other markets consistently exceed 0.3 for the whole period. Also, the correlation between the US (S&P 500) and the UK (FTSE 100) has the strongest positive correlation, showing that these two markets have notably surged to 0.60, and the UK has a moderate positive correlation with South Korea. The enhanced correlations demonstrate a heightened and durable synchronization during this timeframe. However, it is necessary to note that correlation coefficients deviate slightly from tail dependence due to nonlinearity in extreme events.

**Table 4:** Correlation coefficient.

	SP500	HANGSENG	FTSE100	SSEComp	KOPSI
SP500	1				
HANGSENG	0.229806	1			
FTSE100	0.600566	0.417289	1		
SSEComp	0.141415	0.563245	0.216236	1	
KOPSI	0.249639	0.622206	0.389754	0.336584	1

**Note:**Table 4 presents the linear interdependence observed within the stock markets. Notable variations in correlation coefficients are identified. Notably, except for the US with China, Hong Kong & South Korea, and China with the UK, the correlations linking other markets consistently exceed 0.3 for the whole period.

## 5 Empirical results

### 5.1 EGARCH model estimates

The empirical research findings in Table 5 show the EGARCH model's estimations with stock market jumps. The measure shows the persistence of volatility. In general, it is near to 1; however, when the structural breakdowns are included in the models, it decreases slightly. For instance, higher standard deviations and negative skewness might indicate more volatile and negatively skewed returns. The parameter γ shows negative values for China, the UK, and Hong Kong, which indicates unfavorable news effects with more volatility and reflects negatively skewed returns with a solid impetus to the stock market. Furthermore, Q2 (20) and ARCH (20) tests show that Hong Kong has a greater p-value, indicating that only Hong Kong data is consistent with the model. While the remaining indices show a lack of fit between the model and the data.

**Table 5:** Projected parameters of EGARCH.

	SP500	HangSheng	FTSE100	SSEComp	Kopsi
μ	9.10E-04	4.23E-04	3.78E-04	2.12E-04	3.18E-04
ῥ	−3.20E-01	4.22E-01	9.49E-01	3.35E-01	−6.86E-01
Γ	2.43E-01	−4.12E-01	−9.70E-01	−3.18E-01	6.70E-01
Ω	4.21E-06	3.25E-06	5.81E-06	1.55E-06	3.21E-06
α	1.70E-01	6.25E-02	1.26E-01	6.22E-02	8.91E-02
β	8.00E-01	9.19E-01	8.18E-01	9.31E-01	8.81E-01
X-squared (Q20)	197.6	16.245	53.182	41.876	48.253
p-value	2.20E-16	0.7013	7.64E-05	0.00287	0.000392
X-squared (Arch20)	118.23	17.124	51.149	41.397	43.643
p-value	5.55E-16	0.6449	0.0001513	0.003312	0.00168

**Note:**Table 5 shows the EGARCH model's estimations with market jumps. The parameter Γshows negative values for China, the UK, and Hong Kong, which indicates unfavorable news effects with more volatility and reflects negatively skewed returns with a solid impetus to the stock market.

The POT approach models extreme returns using the filtered residuals. A correct threshold selection is crucial for an excess distribution. The estimation bias can be decreased by raising the threshold. The observations must be larger than this threshold to reduce the variance of the estimation when using the data to estimate the relevant parameters.

Our findings indicate that almost all downside levels exceed the upside for the predicted parameters presented in Table 6. The conclusion indicates a potential prevalence of price slumps. To prevent incorrect specification of the copula model, Patton (2006) [50] made the case that information modified by the probability integral should be dispersed evenly. The KS test is used to investigate the inversed data. Consequently, the results do not disprove the uniform hypothesis.

**Table 6:** EVT estimation.

	SP500	HangSheng	FTSE1000	SSEComp	Kopsi
**Lower-Tail**					
Threshold	−2.056950236	−2.435730952	−1.8386	−2.3838	2.145926398
Shape	−0.062974486	−0.096117524	−0.1508	−0.1237	−0.128749725
Local Parameter	0.129334868	0.163294488	0.13154	0.15198	0.139351571
**Upper-Tail**					
Threshold	0.451255134	0.007458643	0.00178	0.30555	0.260374192
Shape	6.34E-06	0.001810437	0.01725	0.02497	3.14E-05
Local Parameter	0.006367392	0.008997638	0.00862	0.0075	0.006686118
KS	0.00532114	0.00584698	0.00612	0.00741	0.00820624

**Note:**Table 6 presents the EVT estimates with the threshold parameters, shape, local parameters, and KS estimates. The KS test is used to investigate the inversed data. We discover that nearly all downside levels are larger than the upside for the predicted parameters, so there may be too many price slumps.

### 5.2 The C-vine Copula quantile regression

We have analyzed to determine the interdependence patterns between stock markets during two distinct eras, utilizing data from the Probability Integral Transform (PIT). Table 7 presents the estimated copulas, which provide insights into unconditional and conditional reliance between these markets. Copula differences, we have predicted further validate the variability in dependency structures across these two eras. During the observation period, our analysis indicates that the Hang Seng Index (Hong Kong) and the KOSPI Composite Index (South Korea) exhibit the strongest dependency relationship, as revealed by the copula values. It is significant to observe that the conditional dependency and direct dependency of the South Korean market on the Hong Kong market exhibit a positive correlation during this timeframe.

**Table 7:** The copulas' estimation outcomes.

	SP500/HangSheng	SP500/FTSE100	SP500/SSEComp	SP500/Kopsi	HangSheng/FTSE100	HangSheng/SSEComp	HangSheng/Kopsi	FTSE100/SSEComp	FTSE100/Kopsi	SSEComp/Kopsi
Whole Period										
Conditional Dependency (Normal Copula)										
Par	0.2496825	0.5695873	0.1409159	0.2568295	0.4111153	0.5820149	0.6183914	0.2156435	0.3568638	0.3565698
Var.Est	0.000205073	0.000111874	0.000286475	0.000208597	0.000184026	0.000130237	9.72E-05	0.000264333	0.000211079	0.000221896
liklihood	91.58391	559.588	28.52055	97.09209	263.758	590.2125	687.9536	67.73625	193.9727	193.6298
Conditional Dependency (Student t Copula)										
Par	0.282790038	0.62654603	0.15500749	0.28251245	0.45222683	0.64021639	0.68023054	0.23720785	0.39255018	0.39222678
Var.Est	0.000442234	0.000123062	0.000315122	0.000229456	0.000202428	0.000143261	1.07E-04	0.000290766	0.000232187	0.000244086
Liklihood	174.04222	615.5468	31.372605	106.801299	290.1338	649.23375	756.74896	74.509875	232.76724	212.99278
Direct Dependency (Normal Copula)										
Par	0.2242402	0.5591014	0.1278428	0.2269918	0.4050494	0.5810732	0.6187169	0.2117278	0.3516017	0.3565367
Var.Est	0.000212331	0.00011561	0.000288472	0.000215031	0.000182908	0.00013103	9.71E-05	0.000262718	0.000206901	0.000221653
liklihood	73.39239	534.7516	23.43117	75.25488	255.2817	587.8453	688.8869	65.24003	187.8945	193.5906
Conditional Dependency (Student t Copula)										
Par	0.2287255	0.61501154	0.143183936	0.26104057	0.450009883	0.63918052	0.68058859	0.23290058	0.38676187	0.39219037
Var.Est	0.000412525	0.000127171	0.000320203	0.000236534	0.000202205	0.000145443	1.07E-04	0.00028899	0.000227592	0.000243819
liklihood	138.6648	588.22676	26.2429104	82.780368	283.7200814	646.62983	702.664638	71.764033	189.773445	212.94966

Note: Table 7 presents the estimated copulas, which provide insights into unconditional and conditional reliance between these markets. Copula differences, we have predicted further validate the variability in dependency structures across these two eras. During the observation period, our analysis indicates that Hong Kong and South Korea exhibit the most robust dependency relationship, as revealed by the copula values. Notably, we find that the South Korean market's conditional and direct dependency on the Hong Kong market positively correlated throughout this period.

The direct dependencies between markets remain consistent with those observed during the period of significant volatility within the South Korean stock market. However, it's worth noting that the direct dependency of the Hong Kong stock market during this high-volatility period exhibits slight variations compared to the conditional dependency observed in a normal copula distribution. We observe stronger dependencies between the Hong Kong market and the China and South Korea indices during this period than other market pairs. It's important to highlight that the tail-distribution behavior in the high-volatility period of the South Korean market closely resembles that of the entire observation period.

During times of high volatility in the Chinese stock market, we observe the strongest interdependence between the stock markets of the United States and the United Kingdom. Similarly, during periods of elevated volatility in the United States, the relationship between the stock markets of Hong Kong and China exhibits the highest level of dependency. In contrast, when the United Kingdom experiences high volatility, the stock markets of Hong Kong and South Korea display the strongest interconnection, resembling the patterns observed during the entire period (refer to Appendix I).

Essential data on conditional reliance substantiate the correlation between Hong Kong's stock market and other global markets. In alternative scenarios, the predicted copulas for the entire study period markedly differ from those observed during high-volatility periods in other stock markets. Larger correlation values signify a more pronounced direct dependency. Additionally, the conditional copulas, computed using the C-vine copula, reveal a link between the U.S. market, Hong Kong, and the Chinese market during the high-volatility periods. These findings diverge significantly from the patterns seen throughout the Chinese period, highlighting the unique nature of the high-volatility phase experienced by the U.S.

During periods of high volatility, these anticipated variations in the stock markets of China, Hong Kong, South Korea, the United Kingdom, and the United States underscore their distinct reliance on other global markets. These observations offer valuable insights into the potential implications of market interdependence on risk. Consequently, the oversight and regulation of risk contagion should extend beyond domestic financial markets, encompassing a vigilant monitoring of relevant global markets.

## 6 Conclusions

This research article investigates volatility dynamics in various stock markets, focusing on the influence of structural breaks and high-volatility periods. The updated ICSS algorithm is utilized to identify key structural junctures in stock market volatility. It employs econometric models like AR (1)-EGARCH(1, 1), POT, and Copula approach to estimate volatility extreme events and dependency patterns during high-volatility periods compared to normal periods, respectively. The article identifies two anomalous periods marked by notable volatility clustering coinciding with global financial crises. Notably, the Chinese stock market crisis in 2014 is highlighted, where the SSEComp index experienced a sharp decline.

There was widespread apprehension when the COVID-19 pandemic started, and major global markets, especially the US stock exchange, saw a sharp decline. An extensive analysis uncovers a strong link between the Chinese stock market and the Hong Kong stock index, suggesting that the Chinese market may utilize the Hong Kong channel to influence other global markets. The significant correlation between the Chinese and United States markets during times of enhanced market volatility highlights the profound interconnectedness of global markets. This comprehensive study uncovers the complex network of interdependencies between global stock markets, with the United States and China exerting significant influence on the impact of these markets on East Asian markets. Developing thorough risk mitigation measures that include the constantly changing nature of these marketplaces, particularly during times of increased uncertainty and change, is a part of the task. Moreover, this study revealed that specific periods of increased market volatility led to a noticeable pattern in which markets synchronize and participate in bilateral relations, potentially causing a cascading impact. The findings of this academic study have significant relevance for risk management and portfolio optimization. The aforementioned practical insights significantly influence risk management and regulatory systems. This empirical study emphasizes the need to consider the complex network of interconnected global markets when evaluating systemic risk and financial stability. Risk contagion, a substantial threat to the financial system's integrity, can occur when risk management efforts do not consider the consequences of excessive cross-border spillovers.

### 6.1 Theoretical implications

This research enhances the understanding of global market interdependencies, particularly during crises. The findings are aligned with the systematic risk theory which addresses the spillover effect of financial systems across the border. The combination of different models offers a nuanced approach to volatility and could inspire further theoretical work on financial contagion and market behavior. Using Garch model helps to understand the asymmetric theory where the study tries to better address the theory through the cross-market volatility effects. Similarly, the applications of C-vine Copula, EVT, CoVaR, and quantile regression extend the literature in a way to address the complex dependencies of risk path. This method can explain nonlinear trends in extreme conditions. This study also contributes to the asymmetric theory through the development of risk contagion paths across different interdependent markets.

### 6.2 Practical implications

The findings suggest the need for more comprehensive risk management strategies considering global market interdependencies. Regulatory policies should account for these connections, and investors can use these insights to optimize portfolios during volatile periods. The findings of the study emphasize the need of developing risk management practices to control the cross-border spillover effect. Financial institutions should also look into market interdependencies before designing portfolios. As the global financial conditions are reflecting domestic financial markets. Using the Copula, C-vine, and EVT theory, portfolio managers can better assess the financial risk and develop profitable investment portfolios for investors. This study also educates the investors regarding risk calculation. Investors understand that nowadays, financial risk in the domestic market is reflected through the spillover effect of other cross-border markets. Therefore, it is essential to consider cross-border risk contagion paths before investing in any financial market. Overall, the methodology of this study can provide the direction of understanding the systematic risk and complex cross-market conditions.

### 6.3 Limitations and future studies

The study's reliance on specific econometric models may not capture all market complexities. Data limitations and the geographical focus on Chinese and East Asian markets might exclude important global interactions. The limited timeframe could also miss broader, long-term trends. Future research could explore volatility dynamics in other regions or conduct longer-term studies to verify observed patterns. Examining non-economic factors and developing alternative modeling approaches could also provide deeper insights into market behavior.

## CRediT authorship contribution statement

**Fahim Afzal:** Writing – review & editing, Writing – original draft, Methodology, Formal analysis, Conceptualization. **Haiying Pan:** Supervision, Resources, Funding acquisition. **Farman Afzal:** Writing – original draft, Conceptualization. **Rana Faizan Gul:** Writing – review & editing.

## Funding

This work was supported by 10.13039/501100009012Hohai University under grant number 2016-423317.

## Data availability statement

The data will be made available upon request.

## Declaration of competing interest

The authors declare that they have no known competing financial interests or personal relationships that could have appeared to influence the work reported in this paper.

## Figures and Tables

**Table:** 

Direct Dependency										
**Normal Copula**										
Par	0.2225761	0.5876196	0.1367036	0.1609179	0.4523542	0.5383563	0.52043	0.1883355	0.3299109	0.2029641
Var.Est	0.001596663	0.000473136	0.001580128	0.001603543	0.000790159	0.000959322	0.0006288	0.001244753	0.001382853	0.001444707
Likelihood	12.17335	102.9413	4.511028	6.276545	55.30825	82.88746	76.53604	8.645105	27.70308	10.0736
**Student t Copula**										
Par	0.1910113	0.64638156	0.153108032	0.180228048	0.506636704	0.602959056	0.5828816	0.21093576	0.369500208	0.227319792
Var.Est	0.003009192	0.000529912	0.001769743	0.001795968	0.000884978	0.001074441	0.000704256	0.001394123	0.001548795	0.001618072
Likelihood	22.06878	115.294256	5.05235136	7.0297304	61.94524	99.39552	85.7203648	9.6825176	31.0274496	11.282432

**Table:** 

Direct Dependency										
**Normal Copula**										
Par	0.09414884	0.4062118	0.05364409	0.1876496	0.3225274	0.6868588	0.5823065	0.2128689	0.307377	0.3954642
Var.Est	0.002484245	0.001758176	0.002863941	0.002258228	0.001983838	0.00073362	0.000847966	0.002817339	0.001839651	0.001705439
liklihood	1.448118	29.69294	0.4684544	5.843967	18.00677	106.8926	68.9151	7.566275	16.25801	27.98607
**Student t Copula**										
Par	0.09972908	0.456582063	0.060295957	0.21091815	0.362520798	0.772029291	0.654512506	0.239264644	0.345491748	0.444501761
Var.Est	0.003479861	0.00197619	0.00321907	0.002538248	0.002229834	0.000824588	0.000953113	0.003166689	0.002067768	0.001916913
liklihood	3.382093	33.37486456	0.526542746	6.568618908	20.23960948	120.1472824	77.4605724	8.5044931	18.27400324	31.45634268

**Table:** 

Direct Dependency										
**Normal Copula**										
Par	0.413116	0.6762576	0.3243419	0.370487	0.5897791	0.6720145	0.7941318	0.3284437	0.5314879	0.591237
Var.Est	0.002844224	0.001619811	0.00412335	0.002628081	0.002028628	0.001524207	0.000839996	0.004933654	0.001747024	0.001732751
liklihood	16.48143	55.27875	9.711871	12.9527	38.31471	54.30331	91.18281	9.978561	29.57762	38.5576
**Student t Copula**										
Par	0.3916764	0.6413324	0.3099155	0.3678865	0.5845982	0.6575018	0.7898522	0.3231042	0.5295287	0.5892728
Var.Est	0.005961826	0.003575009	0.005769034	0.006528723	0.002977275	0.002898949	0.00114535	0.005410513	0.004580538	0.002706682
liklihood	19.77074	60.27118	10.6125	23.84248	40.0678	57.12208	92.86775	10.01235	39.04902	40.33123

**Table:** 

Direct Dependency										
**Normal Copula**										
Par	0.2830663	0.5044051	0.2105928	0.2640822	0.4315538	0.6545711	0.6542435	0.2762137	0.3826383	0.4667466
Var.Est	0.000732007	0.000469434	0.000897685	0.000623049	0.000705929	0.000335484	0.000351775	0.001006269	0.000587932	0.000662113
liklihood	37.00187	130.7609	20.0717	32.01569	91.61527	250.1657	249.829	35.15103	70.28936	109.3249
**Student t Copula**										
Par	0.2778248	0.4804721	0.2076345	0.2554349	0.4210358	0.6537933	0.6446129	0.2723281	0.3649866	0.463865
Var.Est	0.001262333	0.001130179	0.00120331	0.001467836	0.001035627	0.000449726	0.00057089	0.001116229	0.00132878	0.000813126
liklihood	50.0272	176.7346	23.79082	73.06269	97.89394	253.9468	261.8024	35.444	102.1895	110.5393

**Table:** 

Direct Dependency										
**Normal Copula**										
Par	0.3628501	0.621989	0.2845418	0.3226841	0.5235648	0.6582965	0.7545187	0.294294	0.4695643	0.573964
Var.Est	0.002320547	0.001424475	0.0030306	0.002055505	0.001923921	0.001179713	0.00069448	0.00355942	0.001644386	0.00128457
liklihood	17.67043	62.40857	10.51725	13.72723	40.56388	72.67238	108.5826	11.29084	31.41115	50.79522
**Student t Copula**										
Par	0.3436953	0.5642331	0.2701553	0.317234	0.5164306	0.646657	0.749726	0.2863974	0.4599133	0.5733777
Var.Est	0.004432683	0.003711142	0.004271104	0.005023727	0.002692732	0.002051135	0.001059389	0.004035885	0.003970047	0.002036131
liklihood	21.98193	72.10541	12.02767	28.38106	42.25473	75.83024	112.239	11.41381	40.85813	53.45919
